# Toxicity and Binding Studies of Bacillus thuringiensis Cry1Ac, Cry1F, Cry1C, and Cry2A Proteins in the Soybean Pests Anticarsia gemmatalis and Chrysodeixis (Pseudoplusia) includens

**DOI:** 10.1128/AEM.00326-17

**Published:** 2017-05-17

**Authors:** Yolanda Bel, Joel J. Sheets, Sek Yee Tan, Kenneth E. Narva, Baltasar Escriche

**Affiliations:** a Department of Genetics, Universitat de València, Burjassot, Spain; b Estructura de Recerca Interdisciplinar en Biotecnologia i Biomedicina (ERI BioTecMed), Universitat de València, Burjassot, Spain; c Dow AgroSciences, Indianapolis, Indiana, USA; University of Georgia

**Keywords:** Cry proteins, heterologous competition, soya pest, soybean looper, velvetbean caterpillar

## Abstract

Anticarsia gemmatalis (velvetbean caterpillar) and Chrysodeixis includens (soybean looper, formerly named Pseudoplusia includens) are two important defoliating insects of soybeans. Both lepidopteran pests are controlled mainly with synthetic insecticides. Alternative control strategies, such as biopesticides based on the Bacillus thuringiensis (Bt) toxins or transgenic plants expressing Bt toxins, can be used and are increasingly being adopted. Studies on the insect susceptibilities and modes of action of the different Bt toxins are crucial to determine management strategies to control the pests and to delay outbreaks of insect resistance. In the present study, the susceptibilities of both soybean pests to the Bt toxins Cry1Ac, Cry1Fa, Cry1Ca, and Cry2Aa have been investigated. Bioassays performed in first-instar larvae showed that both insects are susceptible to all these toxins. Competition-binding studies carried out with Cry1Ac and Cry1Fa ^125^-iodine labeled proteins demonstrated the presence of specific binding sites for both of them on the midgut brush border membrane vesicles (BBMVs) of both A. gemmatalis and C. includens. Competition-binding experiments and specific-binding inhibition studies performed with selected sugars and lectins indicated that Cry1Ac and Cry1Fa share some, but not all, binding sites in the midguts of both insects. Also, the Cry1Ac- or Cry1Fa-binding sites were not shared with Cry1Ca or Cry2Aa in either soybean pest. This study contributes to the knowledge of Bt toxicity and midgut toxin binding sites in A. gemmatalis and C. includens and sheds light on the cross-resistance potential of Cry1Ac, Cry1Fa, Cry1Ca, and Cry2Aa Bt proteins as candidate proteins for Bt-pyramided crops.

**IMPORTANCE** In the present study, the toxicity and the mode of action of the Bacillus thuringiensis (Bt) toxins Cry1Ac, Cry1Fa, Cry1Ca, and Cry2Aa in Anticarsia gemmatalis and Chrysodeixis includens (important defoliating pests of soybeans) have been investigated. These studies are crucial for determining management strategies for pest control. Bioassays showed that both insects were susceptible to the toxins. Competition-binding studies demonstrated the presence of Cry1Fa- and Cry1Ac-specific binding sites in the midguts of both pests. These results, together with the results from binding inhibition studies performed with sugars and lectins, indicated that Cry1Ac and Cry1Fa share some, but not all, binding sites, and that they were not shared with Cry1Ca or Cry2Aa in either soybean pest. This study contributes to the knowledge of Bt toxicity in A. gemmatalis and C. includens and sheds light on the cross-resistance potential of Cry1Ac, Cry1Fa, Cry1Ca, and Cry2Aa Bt proteins as candidate proteins for Bt-pyramided crops.

## INTRODUCTION

The velvetbean caterpillar Anticarsia gemmatalis Hübner (Lepidoptera: Noctuidae) and the soybean looper Chrysodeixis includens Walker (formerly named Pseudoplusia includens) (Lepidoptera: Noctuidae) are two important defoliating insect pests of soybeans. Indeed, A. gemmatalis is considered one of the main pests of soybean cultivation in South America. These two species usually occur in crops at the same time and can cause great economic damage in soybean-producing regions (1–3).

Soybeans are an important crop that has been increasingly planted (111 million hectares in 2015) (4) and is grown in monoculture in large areas in North and South America. The major soybean-producing nations are the United States, Brazil, and Argentina; these three countries account for more than 80% of the world's supply (CDP, USDA [https://apps.fas.usda.gov/psdonline/circulars/production.pdf]; reference 5). In the United States, the majority of soybeans are grown in the north central states, and the remaining soybeans are grown in the southeastern and Mississippi delta region. Insect pressure is generally greatest in the southeast, where climate allows the major soybean insect pests to overwinter and facilitates multiple generations per year (1). In addition, C. includens can become an important pest of other crops, such as cotton, in the southern areas (6, 7) and in South American countries (3).

A. gemmatalis and C. includens in soybeans have been controlled mainly with synthetic insecticides. Alternative control strategies, such as the use of biopesticides based on Bacillus thuringiensis (Bt), are increasingly being adopted, and transgenic plants expressing Bt toxins have recently been developed as an alternative to control defoliating caterpillars in soybeans (8). Soybeans expressing the Bt toxin Cry1Ac have been commercially available in Brazil since 2013. In 2015, transgenic soybeans with two stacked traits, such as insect resistance due to the expression of Cry1Ac and herbicide tolerance, were grown in 11.9 million hectares in Brazil (4). This stacked soybean event provided high-dose control against A. gemmatalis but not against C. includens (9). Pyramiding Cry1Ac with other Cry toxins from Bt could solve this problem, and more recently, other transgenic soybean events have been developed that produce more than one Cry toxin, such as Cry1Ac and Cry1Fa (10).

In the context of management strategies to ensure pyramided Bt crop durability by delaying insect resistance (defined as a genetically based decrease in susceptibility to a Bt toxin caused by exposure of insects to the toxin [11]), studies on insect susceptibility and mode of action of the different Bt toxins are crucial to determine which insecticidal genes are amenable to pyramiding.

Resistance to Bt proteins could appear if any of the processes involved in their mode of action are disrupted. Although the mode of action of Cry toxins is not completely clarified (12) and the molecular mechanisms underlying these events are not fully understood (13–15), it is known that binding of the Cry toxins to the insect midgut receptors is necessary for toxicity (16–18) and that alterations on binding are commonly associated with high levels of resistance in insect populations (19). Therefore, if different Cry proteins share only a single binding site, an alteration of it could result in cross-resistance, with the consequent loss of effectiveness of the corresponding Bt crop carrying those pyramided Cry proteins.

The use of heterologous competition binding experiments has allowed the development of binding site models that help to optimize the combinations of diverse Cry toxins for increasing the combined pest spectrum and delaying the evolution of resistance to Bt crops. Recent reviews of published binding information for diverse Cry toxins in some relevant Bt-targeted pests enable specific binding site models to be proposed (20, 21). These studies have shown that, in general, (i) Cry1Ac, Cry1Fa, and other Cry proteins can share binding sites in lepidopteran midguts, but the extent and characteristics of shared binding differ among lepidopteran insect species, and that (ii) Cry1C and Cry2 proteins do not share binding sites and do not compete for binding with Cry1A or Cry1F. Some of these binding sites may have glycosylated residues that can be important for the binding interaction (15, 22, 23).

In the present study, the susceptibilities of A. gemmatalis and C. includens to Cry1Ac, Cry1Fa, Cry1Ca, and Cry2Aa were investigated. Bioassays showed that both soybean pests are highly susceptible to the tested Bt toxins. Homologous competition binding studies enabled a determination of Cry1Ac and Cry1Fa quantitative binding parameters, and the heterologous binding studies with Cry1Fa, Cry1Ac, Cry1Ca, and Cry2Aa, together with the study of the inhibitory effect of selected sugars and lectins on Cry1Ac and Cry1Fa binding, helped provide a binding site model for these toxins in A. gemmatalis and C. includens. These studies provide important and necessary information for predicting the benefits of combining the Bt proteins to control A. gemmatalis and C. includens, assessing the resistance and cross-resistance risks associated with the protein combinations, and improving, therefore, control strategies for these pests in the field.

## RESULTS

### Susceptibility of A. gemmatalis and C. includens to Cry proteins.

The results of the analyses of the bioassays conducted with A. gemmatalis and C. includens to assess the toxicity of Cry1Ac, Cry1Fa, Cry1Ca, and Cry2Aa proteins are summarized in Table 1. These insects are highly susceptible to the four Cry proteins, with 50% lethal concentrations (LC_50_s) ranging from 2 to 87 ng/cm^2^.

**TABLE 1:** Toxicity of Bt Cry proteins to neonate larvae of A. gemmatalis and C. includens^*a*^

Protein	A. gemmatalis			C. includens		
	Mortality		GI_50_ (FL_95%_) slope	Mortality		GI_50_ (FL_95%_), slope
	LC_50_ (FL_95%_), slope	LC_90_ (FL_95%_)		LC_50_ (FL_95%_), slope	LC_90_ (FL_95%_)	
Cry1Ac	2.0 (0.9–3.3), 0.9 ± 0.2	23 (13–58)	<1.4	31 (23–41), 1.6 ± 0.2	119 (81–215)	8.9 (4.9–16.2), 1.4 ± 0.5
Cry1Fa	4.9 (3.7–6.6), 1.8 ± 0.3	17 (12–31)	<1.4	4.7 (3.5–6.1), 1.9 ± 0.3	15 (11–27)	<1.4
Cry1Ca	8.9 (6.7–11.6), 1.3 ± 0.1	49 (34–82)	<1.4	3.8 (2.7–5.1), 1.2 ± 0.2	23 (16–40)	<1.4
Cry2Aa	87 (65–116), 1.1 ± 0.1	607 (404–1,060)	<1.4	9.1 (6.9–11.8), 1.3 ± 0.1	47 (33–79)	2.0 (1.6–2.5), 2.6 ± 0.6

a Concentrations are expressed as ng/cm^2^. LC_50_, 50% lethal concentration; FL_95%_, fiducial limits at the 95% level; LC_90_, 90% lethal concentration; GI_50_, 50% growth inhibition. The GI_50_ and slope were not calculated when the growth inhibition response was >50% at the lowest tested rate of 1.4 ng/cm^2^.

Cry1Fa exhibited about the same levels of toxicity for both soybean pests (LC_50_, 4.9 and 4.7 ng/cm^2^ for A. gemmatalis and C. includens, respectively). Cry1Ac was about 15 times more lethal for A. gemmatalis (LC_50_, 2.0 ng/cm^2^) than for C. includens (LC_50_, 31 ng/cm^2^). The Cry1Ca and Cry2Aa proteins were more lethal to C. includens than to A. gemmatalis (2.3 and 9.6 times more, respectively, based on LC_50_).

The mortality response to increased doses of toxin is indicated by the slope parameter in each bioassay. The observed slope values for Cry1Ac and Cry1Fa were 1.6 and 1.9, respectively, for C. includens, and the slope was 1.8 for A. gemmatalis intoxicated with Cry1Fa. The mortality response slope for A. gemmatalis intoxicated with Cry1Ac was about half of that with Cry1Fa (0.9 and 1.8, respectively). Therefore, while the LC_50_ of Cry1Ac in A. gemmatalis showed that this insect was 15 times more susceptible than C. includens to Cry1Ac, the ratio lowered to about 5 times more susceptible when the sensitivity of both insect species was compared at the LC_90_ dose. The Cry1Ca and Cry2Aa slope values for the two insects were very similar, ranging from 1.1 to 1.3.

Growth inhibition observations of both A. gemmatalis and C. includens supported high sensitivity to Cry1Ac, Cry1Fa, Cry1Ca, and Cry2Aa (50% growth inhibition [GI_50_] values, ≤8.9 ng/cm^2^).

### Binding assays with ^125^I-labeled Cry1Ac and ^125^I-labeled Cry1Fa to A. gemmatalis and C. includens BBMVs.

**FIG 1:**
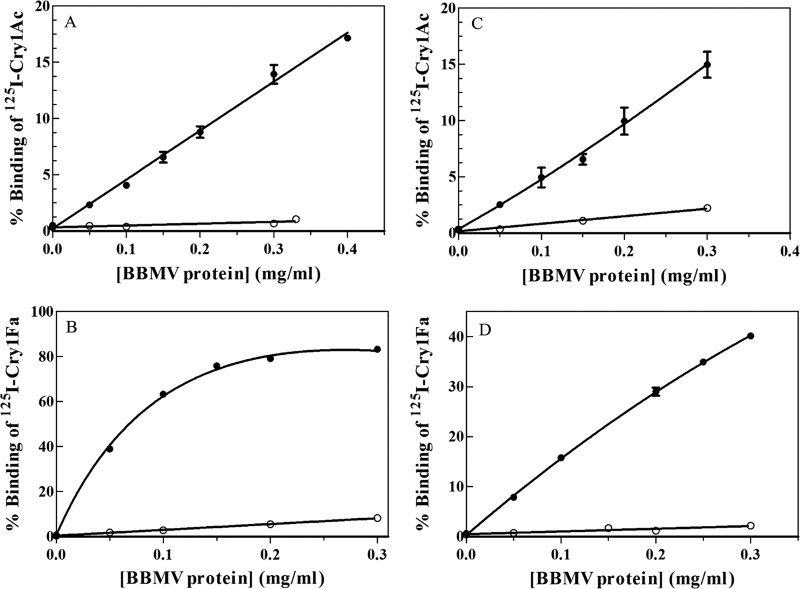
Binding of Cry1Ac and Cry1Fa at increasing concentrations of BBMV proteins. (A to D) A. gemmatalis (A and B) and C. includens (C and D). ●, total binding; ○, nonspecific binding. Results represent the mean and standard deviation of the results from one to two replicates, with several duplicate points.

The Cry1Ac- and Cry1Fa-specific binding activities to A. gemmatalis and C. includens BBMVs were analyzed with increasing BBMV concentrations in the binding reactions. Nonspecific binding was determined using an excess of nonlabeled protein. In all experiments, we obtained specific binding (Fig. 1), with values ranging from 15% specific binding of Cry1Ac in both insect species to 80% binding observed for Cry1Fa with A. gemmatalis BBMV. Based on these experiments, the BBMV concentration for competition binding experiments was determined for each toxin and insect species and 0.05 mg/ml for A. gemmatalis Cry1Fa binding, 0.1 mg/ml for C. includens Cry1Fa binding, and 0.2 mg/ml for A. gemmatalis and C. includens Cry1Ac binding.

### Competitive binding of ^125^I-labeled Cry1Ac to A. gemmatalis and C. includens BBMVs.

**FIG 2:**
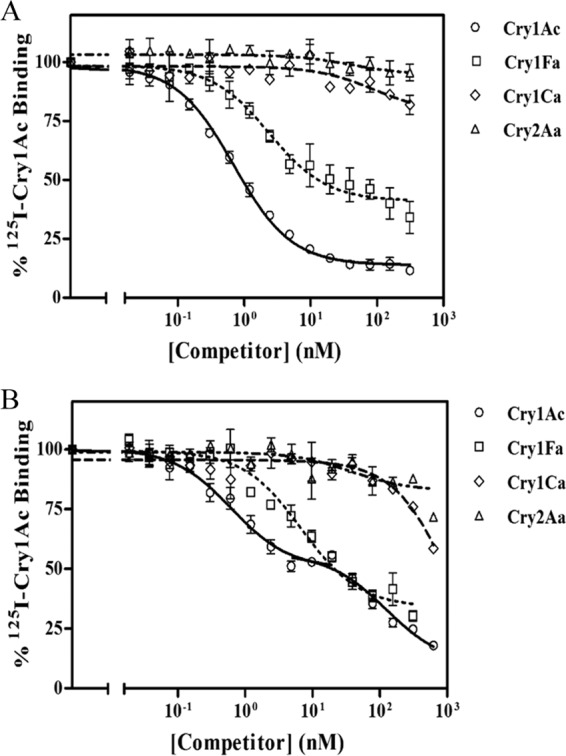
Competition-binding experiments with ^125^I-labeled Cry1Ac. Curves represent total binding of ^125^I-labeled Cry1Ac at increasing concentrations of unlabeled competitor, using BBMV from A. gemmatalis (A) or from C. includens (B). Points represent mean and standard error of the results from three to nine replicated experiments.

Competition experiments were performed by incubating the insect BBMV with ^125^I-labeled Cry1Ac in the presence of increasing amounts of nonlabeled Cry1Ac (for homologous competition experiments) or Cry1Fa, Cry1Ca, and Cry2Aa (for heterologous competition assays). Figure 2 summarizes the results obtained for both insects.

Dissociation constants (*K_d_*) and concentration of binding sites (*R_t_*) were estimated from the homologous competition results (Table 2). Homologous competition data obtained for A. gemmatalis were consistent with the occurrence of a single population of Cry1Ac-binding sites. Cry1Fa partially competed for these binding sites, since labeled Cry1Ac was not displaced at the same rate by unlabeled Cry1Fa as by unlabeled Cry1Ac (Fig. 2A). Cry1Ca and Cry2Aa did not compete for the Cry1Ac-binding sites (Fig. 2).

**TABLE 2:** Binding parameters *K_d_* and *R_t_* calculated from homologous competition assays with BBMV from A. gemmatalis and C. includens^*a*^

Organism	Parameters by toxin			
	Cry1Ac		Cry1Fa	
	*K_d_* (nM)	*R_t_* (pmol/mg of BBMV protein)	*K_d_* (nM)	*R_t_* (pmol/mg of BBMV protein)
A. gemmatalis R1	0.32 ± 0.05	0.33 ± 0.02	5.8 ± 0.8	35 ± 3.2
C. includens R1	0.08 ± 0.12	0.12 ± 0.03	0.48 ± 0.14	3.7 ± 0.3
C. includens R2	13 ± 2.9	23 ± 5.5		

a Results represent the mean ± standard error (SEM) of the results from seven to nine replicates.

For C. includens, the Cry1Ac homologous competition curve fitted a two-site binding model (Fig. 2B), and the analysis of variance (ANOVA) statistical test of the Ligand program supported the hypothesis of two binding sites against the hypothesis of only one, with an *F* value of 52 (*df* = 27, *P* < 0.001): one population had binding sites (receptor 1) with high affinity for Cry1Ac and a low number of binding sites, and another binding site population (receptor 2) had lower affinity for the protein but with higher number of binding sites. Cry1Ca and Cry2Aa did not compete for Cry1Ac sites, while Cry1Fa competed for them, although not completely (Fig. 2B).

### Competitive binding of ^125^I-labeled Cry1Fa to A. gemmatalis and C. includens BBMVs.

**FIG 3:**
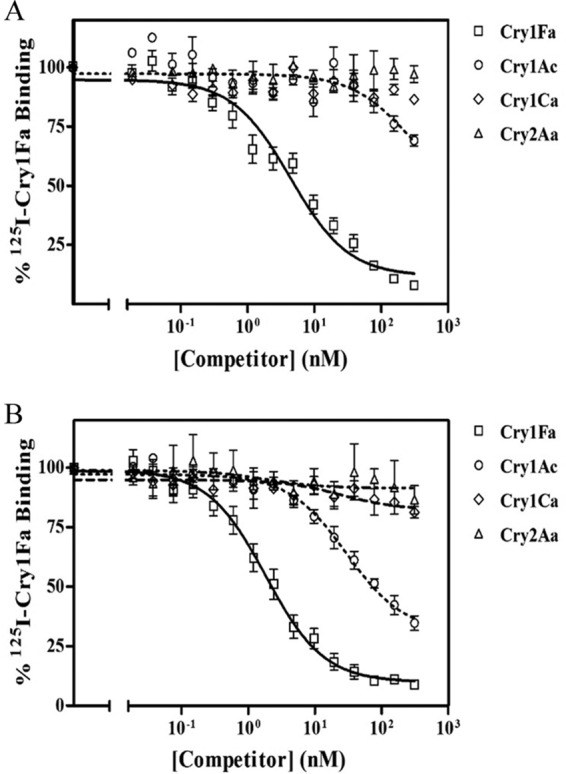
Competition-binding experiments with ^125^I-labeled Cry1Fa. Curves represent total binding of ^125^I-labeled Cry1Fa at increasing concentrations of unlabeled competitor, using BBMV from A. gemmatalis (A) or from C. includens (B). Points represent the mean and standard error of the results from three to eight replicated experiments.

Competition-binding assays with A. gemmatalis and C. includens BBMVs using labeled Cry1Fa protein are shown in Fig. 3A and B, respectively. The binding parameters obtained are summarized in Table 2.

The Ligand analysis fitted the data with the occurrence of a population of binding sites with high affinity for Cry1Fa in both A. gemmatalis and C. includens. Cry1Fa affinity to C. includens binding sites was higher than to A. gemmatalis ones (*K_d_* = 0.48 ± 0.14 nM versus *K_d_* = 5.8 ± 0.8 nM), although C. includens BBMV showed a lower concentration of binding sites (3.7 ± 0.3 pmol/mg BBMV protein versus 35 ± 3.2 pmol/mg BBMV protein). In both insect species, Cry1Ac competed partially and with lower efficiency for these binding sites (Fig. 3). Neither Cry1Ca nor Cry2Aa competed for the Cry1Fa-binding sites.

### Binding inhibition by sugars and lectins.

Different sugars and lectins were tested for their ability to inhibit ^125^I-labeled Cry1Ac and ^125^I-labeled Cry1Fa binding to A. gemmatalis and C. includens BBMVs. The preincubation of the Cry toxins with the sugars and subsequent BBMV binding provide information about the role of BBMV sugars in the interaction with Cry proteins. In addition, the preincubation of lectins with BBMV prior binding to Cry proteins completes this information (24).

**FIG 4:**
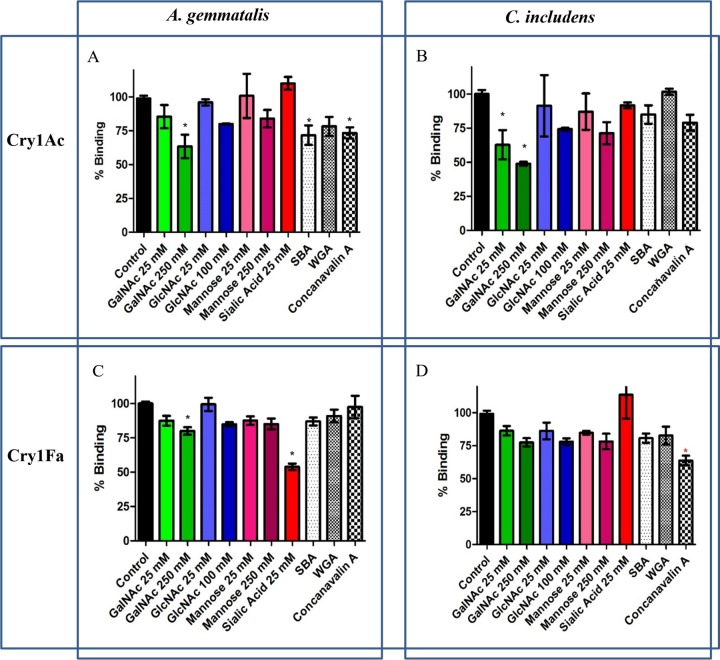
Effect of sugars and lectins on ^125^I-labeled Cry1Ac (A and B) and ^125^I-labeled Cry1Fa (C and D) binding to A. gemmatalis BBMV (A and C) and C. includens BBMV (B and D). The concentration of sugars used in the assays is specified in each bar title on the *x* axis. Bars in the figures represent the standard error of the results from two or three experiments, with replicate points. *, significant differences (*P* < 0.05) in binding with respect to controls, after ANOVA with Tukey's posttest.

The sugars tested were *N*-acetylgalactosamine (GalNAc), *N*-acetylglucosamine (GlcNAc), *N*-acetylneuraminic acid (sialic acid), and α-d-mannose (mannose). The lectins tested were soybean agglutinin (SBA; which binds GalNAc), wheat germ agglutinin (WGA; which binds, GlcNAc and, with lower affinity, sialic acid), and concanavalin A (ConA; which binds mannose and, with lower affinity, glucose) (25). The results pointed to different binding inhibition patterns, depending on the insect species and on the Cry proteins tested (Fig. 4).

Cry1Ac binding to both A. gemmatalis and C. includens BBMVs was negatively affected by preincubation of toxins with GalNAc (Fig. 4A and B). The relevance of GalNAc in binding to A. gemmatalis BBMV was reinforced by the fact that BBMV preincubation with SBA also significantly affected the Cry1Ac binding. In addition, Cry1Ac binding to A. gemmatalis was inhibited about 25% by ConA. No statistically significant differences in binding inhibition by lectins were observed in C. includens, where GalNAc greatly interfered with Cry1Ac binding, indicating an important role of this sugar residue in C. includens BBMV binding sites.

Cry1Fa binding to C. includens BBMV was not blocked by any of the tested sugars (differences were not statistically significant). In A. gemmatalis, sialic acid (and GalNAc, to a lesser extent) significantly impaired Cry1Fa binding to BBMV, reducing it to almost 50% (Fig. 4C and D). However, the reciprocal expected inhibition with WGA (lectin which binds sialic acid) was not observed (Fig. 4C). In fact, none of the tested lectins affected Cry1Fa binding to A. gemmatalis BBMV. On the other hand, Cry1Fa binding to C. includens BBMV was not inhibited significantly by any of the tested sugars, whereas ConA significantly reduced Cry1Fa binding to a level of about 65%.

From the insect perspective, in A. gemmatalis, the binding of both Cry1Ac and Cry1Fa is affected by GalNAc, but lectins, such as SBA and ConA, affect Cry1Ac binding but not Cry1Fa binding. Conversely, sialic acid affected binding of Cry1Fa but not Cry1Ac. In C. includens, GalNAc did not interfere with Cry1Fa binding but did impair Cry1Ac binding. ConA significantly affected Cry1Fa binding but did not affect binding of Cry1Ac. Taken together, and considering the competition binding results, these data indicate that Cry1Ac and Cry1Fa share some but not all BBMV binding epitopes in A. gemmatalis and in C. includens.

## DISCUSSION

The results presented in this work show that B. thuringiensis insecticidal proteins Cry1Ac and Cry1Fa were highly toxic to the two lepidopteran soybean pests A. gemmatalis and C. includens, with LC_50_s ranging from 2.0 to 30.5 ng/cm^2^. Cry1Ac was more active against A. gemmatalis than against C. includens (Table 1). These results are in agreement with previous studies performed using diet surface overlay (26) and by diet incorporation (9, 27, 28). In all cases, the LC_50_s obtained showed that A. gemmatalis was more susceptible to Cry1Ac than was C. includens, by ratios ranging from 16 to 93 times. In our study, performed using diet surface overlay, the toxicity ratio was more than 15 times greater for A. gemmatalis than for C. includens, a value very close to those in previously mentioned reports. The difference in susceptibility has also been observed in studies performed with insecticidal Bt strains, such as HD1 and BC1, where A. gemmatalis was about 5.6 and 1.5 times more susceptible, respectively, than C. includens (29).

To our knowledge, there are no published reports describing Cry1Fa toxicity on A. gemmatalis and C. includens. In the present study, Cry1Fa was shown to be highly toxic to both A. gemmatalis and C. includens, with very similar LC_50_s (about 5 ng/cm^2^). Cry1Fa was 2.5 times less toxic than Cry1Ac to A. gemmatalis, while Cry1Fa was 6.5 times more toxic than Cry1Ac to C. includens.

Cry1Ca and Cry2Aa were more toxic to C. includens than to A. gemmatalis, similar to findings reported by Crialesi-Legori et al. for Cry1Ca (26), who reported LC_50_ values for Cry1Ca slightly higher than the ones obtained in this study. On the other hand, it has been described that Cry2A was either nontoxic to A. gemmatalis (30) or toxic when tested by diet incorporation (31, 32). Our results support the high toxicity of Cry2Aa, especially for C. includens larvae.

Toxin binding studies with each insect species provide information on the potential for changes in binding to cause cross-resistance among the toxins. Several studies performed with lepidopteran insects resistant to Cry1Ac or to Cry1F have shown that cross-resistance, expressed as a resistance ratio, can range from absent to slight or high, depending on the species considered. The Cry1Ac-resistant colonies YHD2 of Heliothis virescens, SZBT of Plutella xylostella, or ACB-AcR of Ostrinia furnacalis showed moderate to high cross-resistance to Cry1Fa (33–35). However, the GLEN-Cry1Ac-BCS colony of Trichoplusia ni, the Helicoverpa zea GA-R selected strain, and the BtR-resistant strain of Helicoverpa armigera showed low or slight cross-resistance to Cry1F (36–38). On the other hand, an Ostrinia nubilalis Cry1F selected strain and several Spodoptera frugiperda Cry1F-field-resistant strains (field strains or derived from the field-resistant strains) showed slight cross-resistance to Cry1Ac (39–43). The exceptions were some S. frugiperda isofamilies derived from the Puerto Rico Cry1F field-tolerant insects, which developed moderate tolerance to Cry1Ac (44).

In this paper, we have analyzed the quantitative binding of Cry1Ac and Cry1Fa in A. gemmatalis and in C. includens BBMV to evaluate the cross-resistance potential associated with their combination in transgenic soybeans expressing these proteins, as well as their cross-resistance potential with Cry1Ca and Cry2Aa. The competitive binding results showed that in A. gemmatalis, Cry1Fa could compete for some, but not all, of the Cry1Ac-binding sites (Fig. 2A), and that Cry1Ac competed only at high concentrations for Cry1Fa-binding sites (Fig. 3A). In C. includens, the results showed that Cry1Ac bound to two different binding site populations, with different affinities (Table 1), and Cry1Fa competed for part of those binding sites (Fig. 2B). Cry1Ac also competed with Cry1Fa for a portion of the binding sites in C. includens (Fig. 3B). At this point, it is interesting to keep in mind that the binding sites revealed by the competition experiments could be composed of several populations of molecules with similar binding affinities that cannot be distinguished by BBMV competition binding experiments. Cry1Ca and Cry2Aa did not compete for Cry1Ac- or Cry1Fa-binding sites in A. gemmatalis or in C. includens. These binding site competition data provide a binding site model that is similar for A. gemmatalis and C. includens, in which Cry1Ac and Cry1Fa would have their own nonshared binding sites in addition to other shared receptors, and in which Cry1Ca and Cry2Aa would not share any of these binding sites.

The binding site model obtained for A. gemmatalis and C. includens is, in general, similar to those proposed for some of the most relevant Bt-targeted lepidopteran pests in the last years (e.g., P. xylostella, O. nubilalis, Spodoptera spp., and heliothines, such as H. virescens, H. zea, and H. armigera) (20, 21). The binding site models suggest that, depending on the lepidopteran species, Cry1Ac and Cry1Fa may have several binding sites with different patterns of toxin interaction, and Cry1Ca and Cry2Aa do not compete for these sites, nor do they compete with one another. The only exceptions are Spodoptera exigua and S. frugiperda, where Cry1Fa was reported to compete with labeled Cry1Ca in one study (17), whereas in a more recent work performed with S. frugiperda, it was found that Cry1Fa and Cry1Ca did not compete with each other (45). This recent study finding is similar to what had been found for Cry1Fa and Cry1Ca binding in other insects and what we found for A. gemmatalis and C. includens in the present work.

Some of the Cry1-binding sites may have glycosylated residues that may be involved in the binding interaction (24, 46–48). To determine if sugars were a common characteristic in the binding of Cry1Ac and Cry1Fa to A. gemmatalis or to C. includens, different sugars and lectins were tested for their ability to inhibit ^125^I-labeled Cry1Ac and ^125^I-labeled Cry1Fa binding to the BBMVs. Indeed, in H. virescens, altered glycosylation correlated with reduced Cry1A and Cry1Fa binding (47), and the glycosylated protein was identified as an alkaline phosphatase (ALP) that served as a Cry1Ac receptor in H. virescens (49). Later, a similar result was obtained in H. armigera (50). Also, a GalNAc residue in a 120-kDa aminopeptidase-*N* (APN) receptor has been described as being involved in Cry1Ac binding in Manduca sexta (46). The involvement of sugar residues in Cry toxin binding can be assessed by binding inhibition experiments (24). Using this approach, our results pointed to an important role of GalNAc residues for Cry1Ac binding to both A. gemmatalis and C. includens BBMVs (Fig. 4A and B). In accordance with this, SBA should inhibit Cry1Ac binding, since this lectin has high affinity by GalNAc moieties (25). This was observed indeed in A. gemmatalis, but not in C. includens, where the percentage of binding inhibition was not statistically significant. In addition, ConA inhibited more than 25% Cry1Ac binding in A. gemmatalis, indicating different Cry1Ac-binding site characteristics between the two insects.

In contrast to Cry1Ac, Cry1Fa binding in A. gemmatalis BBMV was not affected by any of the lectins tested, and only sugars, such as sialic acid and GalNAc (at the highest concentration tested), reduced the Cry1Fa binding in about 45% and 20%, respectively (Fig. 4C). In C. includens, none of the sugars affected Cry1Fa binding, but ConA, a lectin that binds specifically to mannose and glucose residues, significantly reduced Cry1Fa binding to about 65% (Fig. 4D). In line with the reasoning proposed by Estela et al. (24), these data might indicate that residues of mannose or glucose can be near the epitopes to which Cry1Fa binds, although these sugars would not be used for Cry binding.

To date, Cry1Ac has been introduced in economically relevant crops, such as cotton and soybeans. It was shown that transgenic soybeans and cotton expressing Cry1Ac alone did not provide full economic control of C. includens (9, 51). The addition of Cry1F in cotton improved the control of this insect, as well as the control of other insect pests, such as S. frugiperda (51).

The results presented in this paper indicate that (i) Cry1Ca and Cry2Aa do not compete for Cry1Ac- or Cry1Fa-binding sites in A. gemmatalis and in C. includens, and this indicates that different pairs of these proteins could be combined in soybean plants as effective pyramids (multiple toxins) to mitigate the potential development of resistant insect populations; and (ii) Cry1Ac and Cry1Fa have independent nonshared binding sites in addition to some shared sites in either of these two insect species. Resistance management for soybeans expressing these two proteins should account for the potential for incomplete cross-resistance between them to develop in the field. If the nonshared binding sites are important in toxin activity, low levels of cross-resistance would be expected, such that the soybeans would have the properties of a true pyramid with redundant killing. This would lead to high durability of the toxins when the soybeans are grown with a small refuge (52). On the other hand, if the shared binding sites solely are relevant to toxin activity, the crops may select for high levels of cross-resistance, and the durability would be more similar to that of single-toxin plants, requiring a larger refuge to achieve the same resistance management benefits. Incomplete cross-resistance, whereby insects with resistance to one toxin remain moderately susceptible to the other, can provide intermediate resistance management benefits.

Further characterization of the receptors involved in Cry toxin binding in each of the two lepidopteran species will enable a more thorough understanding of the potential patterns and extent of cross-resistance and, therefore, the long-term durability of the Cry protein pyramids to control these two soybean pests.

## MATERIALS AND METHODS

### Insects.

A. gemmatalis was obtained from a colony that was started from insects collected from a field in Florida and reared by Benzon Research, Inc. (Carlisle, PA). C. includens was obtained from a laboratory colony at the University of Georgia and reared in-house at Dow AgroSciences LLC. Both A. gemmatalis and C. includens colonies were laboratory-reared for at least 50 and 32 generations, respectively, prior to insect bioassays.

A. gemmatalis larvae were reared at 29°C, 50% relative humidity (RH), and 16:8 h light/dark with a USDA Stoneville custom-blend artificial diet. The adults were reared at 27°C, 65% RH, and 16:8 h light/dark. Both the larval and adult stages of C. includens were reared at 25°C, 50 to 60% RH, and 16:8 h light/dark, and the larvae were fed the Multispecies Lepidoptera diet with mold inhibitor (Southland Products, AR).

### Bacterial strains and toxin purification.

Cry proteins were expressed in recombinant Pseudomonas fluorescens strains, as described previously (53). Expression of *cry* genes from the *Ptac* promoter was induced by the addition of isopropyl-β-d-1-thiogalactopyranoside (IPTG) after an initial incubation of 24 h at 30°C with shaking in M9 medium supplemented with 1% glucose, trace elements, and 5% glycerol. Cry protein inclusion bodies (IB) were prepared as follows. Bacterial cell pellets were resuspended to 25% (wt/vol) in lysis buffer (50 mM Tris [pH 7.5], 200 mM NaCl, 20 mM EDTA disodium salt [EDTA], 1% Triton X-100, and 5 mM dithiothreitol [DTT], along with 5 ml/liter of bacterial protease inhibitor cocktail (catalog no. P8465; Sigma-Aldrich, St. Louis, MO). The cells were suspended using a hand-held homogenizer (Tissue-Tearor; BioSpec Products, Inc., Bartlesville, OK) at the lowest setting. Lysozyme (0.6 mg/ml; catalog no. L7651; Sigma-Aldrich) was added to the cell suspension by mixing with a metal spatula, and the suspension was incubated at room temperature for 1 h. The suspension was cooled on ice for 15 min and then sonicated using a Branson sonifier 250 (two 1-min sessions, at 50% duty cycle, 30% output). Cell lysis was confirmed by microscopy. The lysate was centrifuged at 11,500 × *g* for 25 min (4°C) to pellet the IBs. The IB pellet was suspended in 100 ml of lysis buffer and homogenized as described above. The IB pellet was then repeatedly washed by suspension in 50 ml of lysis buffer. Finally, the IB pellet was washed and suspended in sterile-filtered (0.22-μm pore size) distilled water containing 2 mM EDTA and pelleted by centrifugation. The final pellet was suspended in sterile-filtered distilled water containing 2 mM EDTA and stored in 1-ml aliquots at −80°C.

For solubilization of IBs, 6 ml of IB suspension was centrifuged to pellet the inclusions. The supernatant was removed, and 25 ml of 100 mM sodium carbonate buffer (pH 11) was added in a 50-ml conical tube. IBs were resuspended using a pipette and vortexed to mix thoroughly. The tube was placed on a gently rocking platform at 4°C overnight to solubilize the Cry protein. The solution was centrifuged at 30,000 × *g* for 30 min at 4°C, and the resulting supernatant was concentrated 5-fold using an Amicon Ultra-15 regenerated cellulose centrifugal filter device (30,000 molecular weight cutoff; Millipore). The sample buffer was then exchanged to 10 mM CAPS [3-(cyclohexamino) 1-propanesulfonic acid] (pH 10), using disposable PD-10 columns (GE Healthcare, Piscataway, NJ).

Cry proteins as IBs were solubilized in buffer containing 20 mM sodium carbonate buffer, 10 mM DTT, and 0.1% 2-mercaptoethanol (pH 11). The solution was centrifuged at 27,000 × *g* for 10 min at 37°C to remove insoluble material. The supernatant was retained and adjusted to a pH of 8.0 with HCl. Upon treatment with 0.5% (wt/vol) tosylsulfonyl phenylalanyl chloromethyl ketone (TPCK)-treated trypsin (Sigma), a Cry protein precipitate formed. After 30 min of incubation with stirring, 1 ml of the protease inhibitor cocktail (catalog no. P8849; Sigma) was added, and the solution was put on ice. The solution was centrifuged at 27,000 × *g* for 10 min at 4°C, and the precipitate was suspended into 20 mM Na_2_CO_3_ (pH 9.6) with stirring. After filtration, the solubilized protein was loaded onto a Pharmacia Mono Q 1010 column equilibrated with 20 mM carbonate buffer (pH 9.6). After washing the loaded column with 2 column volumes of buffer, the truncated toxin was eluted with a linear gradient of 0 to 0.6 M NaCl in 20 mM Na_2_C0_3_ (pH 9.6) in 15 column volumes, at a flow rate of 1.0 ml/min. Purity was checked by SDS-PAGE and staining with Coomassie brilliant blue dye. In some cases, the combined fractions of the purified toxin were dialyzed overnight with 20 mM Na_2_CO_3_ (pH 9.6), reloaded onto the Mono Q 1010 column, and purified a second time. Fractions comprising a single peak were combined, dialyzed, and concentrated.

### Bioassays.

Insect bioassays were performed using the diet overlay method described elsewhere (54). The susceptibility to Cry1Ac and Cry1Fa protoxins was tested with neonate larvae (24 to 48 h old) of C. includens and A. gemmatalis. C. includens eggs were supplied in-house at Dow AgroSciences LLC, while the eggs of A. gemmatalis were commercially obtained from Benzon Research, Inc. (Carlisle, PA). Seven different concentrations of each protein, ranging from 1 to 3,000 ng/cm^2^, and 10 mM CAPS (pH 10) negative-control buffer were exposed to these test insects for 5 days. These treatments were tested using 16 larvae per sample and replicated two times.

The total number of insects exposed to each protein sample, the number of dead insects, and the weights of surviving insects were recorded in all insect bioassays. Percent mortality and percent growth inhibition were calculated for each treatment. Growth inhibition (GI) was calculated as follows: GI = [1 − {(TWIT/TNIT)/(TWIBC/TNIBC)}] × 100, where TWIT is the total weight of insects in the treatment, TNIT is the total number of insects in the treatment, TWIBC is the total weight of insects in the buffer control, and TNIBC is the total number of insects in the buffer control. Control mortality did not exceed 20%.

Probit analyses (55) of the pooled mortality data were conducted using POLO-PC (LeOra Software) to estimate the 50% lethal concentration (LC_50_), 90% lethal concentration (LC_90_), and slope of the concentration-response curves. The slope and growth inhibition concentration-response curves were determined by using a nonlinear logistic 3-parameter model, and the effective concentrations required to cause 50% growth inhibition (GI_50_) and 90% growth inhibition (GI_90_) were estimated. These analyses were performed by using the JMP Pro software, version 9.0.3 (SAS Institute, Inc., Cary, NC).

### Midgut isolation and brush border membrane vesicle (BBMV) preparation.

Midguts were dissected from last-instar larvae of C. includens and A. gemmatalis, washed with 9 volumes of ice-cold homogenization buffer (300 mM mannitol, 5 mM EGTA, 17 mM Tris [pH 7.5]), supplemented with protease inhibitor cocktail (catalog no. P-2714; Sigma) diluted as recommended by the supplier, snap-frozen in liquid nitrogen, and preserved at −80°C until required for use.

BBMVs were prepared by the differential magnesium precipitation method (56). The final BBMV pellet was suspended in ice-cold half-strength MET buffer (125 mM mannitol, 8.5 mM Tris, and 2.5 mM EGTA [pH 7.5]). Subsequently, the BBMV suspension was divided into aliquots that were immediately frozen in liquid nitrogen and stored at −80°C until used. Protein concentration was determined by the Bradford protein assay (57) using bovine serum albumin (BSA) as a standard.

To check the quality of the BBMV, we tested the brush border membrane protein enrichment by measuring aminopeptidase-*N* (APN)- and alkaline phosphatase (ALP)-specific activities in several BBMV preparations. Leucyl-*p*-nitroanilide and 4-nitrophenyl phosphate were used as APN and ALP substrates, respectively (58). The membrane protein enrichment ratio was obtained by comparing the AMP and ALP activities in the final BBMV preparations with respect to the ones measured in the initial midgut homogenate. The enrichment values for AMP and ALP activities in C. includens ranged usually from 3 to 5 times and from 4 to 7 times, respectively. For A. gemmatalis BBMV, AMP activity was usually enriched about 1.5 times, and ALP enrichment ranged from 1.2 to 2.4 times.

### Iodination of Cry proteins.

Labeling of purified truncated proteins with Na ^125^I (PerkinElmer, Inc., Billerica, MA) was performed using the chloramine T method (59–61). Proteins (25 μg) were labeled with 0.5 mCi of Na ^125^I. Both Cry1Ac and Cry1F were labeled two times, and homologous and heterologous competition experiments were performed with each batch of labeled protein. The estimated specific activities of the labeled proteins were 6.5 and 5.5 μCi/μg for Cry1Ac and 2.7 and 0.4 μCi/μg for Cry1Fa and were calculated as previously described (60).

### Binding assays.

BBMV suspensions were centrifuged for 10 min at 16,000 × *g* at 4°C and suspended in binding buffer (phosphate-buffered saline [PBS]–0.1% BSA). Binding assays were carried out in a final volume of 0.1 ml. The optimal concentration of BBMV to be used in the competition experiments was determined by incubation of the labeled toxin (either 0.2 nM ^125^I-labeled Cry1Ac or 0.4 to 3 nM ^125^I-labeled Cry1Fa), with increasing amounts of BBMV for 1 h at room temperature. An excess of unlabeled toxin (0.3 μM) was used to assess the nonspecific binding. After incubation, samples were centrifuged at 16,000 × *g* for 10 min. Pellets were washed with 0.5 ml of ice-cold binding buffer. Radioactivity retained in the pellets was measured in a Gamma counter (2480 WIZARD^2^ automatic gamma counter; PerkinElmer, Downers Grove, IL, USA). Each experiment was conducted with several duplicate points and repeated at least two times.

Time course experiments with A. gemmatalis and C. includens BBMVs were performed to determine the optimum reaction times for the binding experiments. The incubation times for A. gemmatalis BBMV were set at 90 min and 50 min for Cry1Ac and Cry1Fa, respectively, and for C. includens BBMV, the incubation times were 40 min and 90 min for Cry1Ac and Cry1Fa, respectively (data not shown).

Homologous and heterologous competition experiments were carried out by adding increasing concentrations of unlabeled proteins to the tubes containing the labeled proteins and the appropriate concentration of A. gemmatalis or C. includens BBMV (0.2 mg/ml A. gemmatalis or C. includens BBMV for ^125^I-labeled Cry1Ac reactions and 0.05 mg/ml A. gemmatalis BBMV and 0.1 mg/ml C. includens BBMV for ^125^I-labeled Cry1Fa reactions) in a final volume of 0.1 ml. Each competition experiment was conducted with several duplicate points and was repeated at least two times. Binding parameters (dissociation constant [*K_d_*] and concentration of binding sites [*R_t_*]) were estimated with the Ligand program (62). Graphic representations and curve fittings were performed with the GraphPad Prism version 5.00 for Windows (GraphPad Software, San Diego, CA).

### Binding inhibition of iodine-labeled proteins by sugars and lectins.

Binding inhibition experiments with sugars and lectins were performed as described by Estela et al. (24). The ability of sugars to bind the Cry proteins and interfere with their subsequent binding to BBMV was assessed by preincubating the Cry proteins with the sugars (45-min incubation at 25°C) before adding the insect BBMV. The capacity of lectins to bind to BBMV components and interfere with the subsequent binding of the Cry proteins was determined by preincubating the BBMV with the lectins for 45 min at 25°C before starting the assay with the addition of the labeled Cry protein.

The sugars tested were *N*-acetylgalactosamine (GalNAc), *N*-acetylglucosamine (GlcNAc), *N*-acetylneuraminic acid (sialic acid), and α-d-mannose (mannose). The lectins tested were soybean agglutinin (SBA), wheat germ agglutinin (WGA), and concanavalin A (ConA). All compounds were purchased from Sigma-Aldrich Co. LLC, St. Louis, MO, USA. The sugar concentrations used for the inhibition experiments were 25 mM and 100 or 250 mM, and for inhibition experiments with lectins, the concentration was 50 μg/ml. Binding inhibition assays were mostly performed with duplicate points and were replicated two to three times.

One-way ANOVA with Tukey's posttest was performed to compare the levels of binding between controls and samples preincubated either with sugars or with lectins. Statistical analyses and graphs were performed using GraphPad Prism version 5.00 for Windows.
